# Spatial multi-omics and single-cell transcriptomics uncover senescence-associated cellular programs during colon adenoma to cancer progression

**DOI:** 10.64898/2026.08.06.743310

**Published:** 2026-09-04

**Authors:** Ming Yu, Yumo Xie, Lena Allen, Kelly Carter, Elizabeth Donato, Lingxin Cheng, Christina Kendziorski, Kristina A Matkowskyj, Angelo M De Marzo, Jessica Hicks, Deepti Reddi, Evan W Newell, Wei Sun, William M Grady

## Abstract

Colorectal cancer develops through a normal-adenoma-carcinoma sequence, yet only 5-10% of adenomas progress to malignancy, and the cellular programs governing that sequence remain poorly defined. Here we generate a spatial multi-omics atlas of human colon adenomas, combining Visium CytAssist and protein co-detection across 24 nonadvanced and advanced tubular adenomas with single-cell resolution Xenium Prime 5K profiling of 101 patient-matched normal, adenoma, and carcinoma cores from 16 patients. Integrating whole-transcriptome and 31-plex protein data identifies nine spatial clusters and two dysplastic epithelial populations that co-express stemness, proliferation, and senescence programs. These programs occupy a shared, spatially confined epithelial niche that expands from adenoma to carcinoma. Spatial analysis revealed GDF15, a senescence-associated secretory factor, mediated the coupling between senescence and stemness in advanced adenomas, and that GDF15-high epithelium locally excludes CD8+ T cells in adenoma and, more broadly, in carcinoma. These findings position senescence as a spatially instructive rather than merely tumor-suppressive program during colorectal carcinogenesis and suggest GDF15 might be a potential candidate target for cancer prevention and interception in the colon.

## Full Text Availability

The license terms selected by the author(s) for this preprint version do not permit archiving in PMC. The full text is available from the preprint server.

